# The Proximate Phonological Unit of Chinese-English Bilinguals: Proficiency Matters

**DOI:** 10.1371/journal.pone.0061454

**Published:** 2013-04-30

**Authors:** Rinus Gerardus Verdonschot, Mariko Nakayama, Qingfang Zhang, Katsuo Tamaoka, Niels Olaf Schiller

**Affiliations:** 1 Graduate School of Languages and Cultures, Nagoya University, Nagoya, Japan; 2 Leiden Institute for Brain and Cognition, Leiden University Centre for Linguistics & Cognitive Psychology Unit, Leiden University, Leiden, The Netherlands; 3 Faculty of Letters, Arts and Sciences, Waseda University, Tokyo, Japan; 4 Key Laboratory of Behavioral Science, Institute of Psychology, Chinese Academy of Sciences, Beijing, PR China; University of Leicester, United Kingdom

## Abstract

An essential step to create phonology according to the language production model by Levelt, Roelofs and Meyer is to assemble phonemes into a metrical frame. However, recently, it has been proposed that different languages may rely on different grain sizes of phonological units to construct phonology. For instance, it has been proposed that, instead of phonemes, Mandarin Chinese uses syllables and Japanese uses moras to fill the metrical frame. In this study, we used a masked priming-naming task to investigate how bilinguals assemble their phonology for each language when the two languages differ in grain size. Highly proficient Mandarin Chinese-English bilinguals showed a significant masked onset priming effect in English (L2), and a significant masked syllabic priming effect in Mandarin Chinese (L1). These results suggest that their proximate unit is phonemic in L2 (English), and that bilinguals may use different phonological units depending on the language that is being processed. Additionally, under some conditions, a significant sub-syllabic priming effect was observed even in Mandarin Chinese, which indicates that L2 phonology exerts influences on L1 target processing as a consequence of having a good command of English.

## Introduction

Speaking involves the retrieval of phonological representations and their conversion into articulatory commands. According to the language production model by Levelt, Roelofs and Meyer [1], an essential step in this process is the *insertion of phonemes into syllabic metrical frames* (called segment-to-frame association). One of the motivations for the construction of syllables in this way comes from frequent re-syllabification found in Dutch and English [1], which necessitates an on-the-fly construction rather than the storage of syllables. The motivation that this process takes places in units of *phonemes* comes from results of implicit priming experiments [2], [3] as well as priming experiments [4]–[8]. The implicit priming paradigm first teaches associations to certain prompt words (e.g. prompt: *single* – response: *loner*). When participants are subsequently prompted (e.g. *single*), they should verbally produce the learned association (e.g. *loner*). By manipulating the responses to the prompts one can create homogenous groups (*loner, local, lotus*) or heterogeneous groups (*loner, bread, car*). It has been shown that when the onset (phoneme or more) overlaps significant facilitation effects occur but not when only the ending overlaps (e.g. *murder, wonder, boulder*). It has been shown that significant facilitation effects occur for homogeneous groups but not for heterogeneous groups. These results were taken to indicate the incremental nature and unit size of the association-to-frame process in the Levelt et al. model [1]. In the same way, masked priming research [4], [7], [9] has shown that when a prime is presented briefly (50 ms) immediately before a to-be-named target (e.g. *pear*), naming latencies are significantly faster when the prime-target pairs share the onset (e.g. *pole-PEAR*) than when the prime-target pairs do not share the onset (e.g., *take-PEAR*).

However, these results were all obtained with Indo-European languages such as Dutch and English. In fact, several previous studies indicate that the size of the unit to fill the metrical frames may be divergent across languages. For instance, Chen, Chen and Dell [10] and, more recently, O'Seaghdha, Chen and Chen [11] pointed out that a language such as Mandarin Chinese has very different phonological properties from Dutch and English. For instance, Mandarin has a limited inventory of syllables; syllables have a very simple structure and re-syllabification is absent. In several implicit priming experiments, Chen et al. [10] and O'Seaghdha et al. [11] reliably showed syllabic priming effects, but did not find onset-priming effects with monolingual Mandarin Chinese speakers (see also Wong, Huang, and Chen [12] who, although observing word-initial body priming in Cantonese Chinese, did not obtain significant onset priming effects for Cantonese either). Similarly, in Japanese, a profoundly mora-based language (usually comprising of a CV or V, e.g. /ka/ or /a/, a nasal coda, /N/, or a geminate /Q/, but never a single consonant, e.g /k/), Kureta, Fushimi, and Tatsumi [13] using the implicit priming paradigm, and Verdonschot et al. [14] using the masked priming paradigm, were unable to observe onset priming effects. In these studies, significant priming was obtained only when the *mora* overlapped within a homogenous group, or between prime-target. To preserve the way in which phonological encoding is defined in the Levelt et al. model [1] but still being able to account for cross-linguistic differences, O' Seaghdha and Chen [15] and O'Seaghdha et al. [11] coined the term *proximate unit* as a unit differing in *grain size* between languages, which is used to fill the metrical frame. In Dutch/English this would be the phoneme, in Mandarin Chinese the syllable (but see [12], [16], [17] who assume a role for sub-syllabic constituents in *Cantonese* Chinese) and in Japanese the mora [13], [14].

This paper is concerned with a consequence of the proximate unit principle. Specifically, we are interested in the situation when a person *speaks more than one language*, particularly in the case when the unit is proposed to differ such as between Mandarin Chinese and English. More specifically, we would like to explore whether the production system can distinguish and/or adjust the grain size depending on the language used and whether the unit size of one language influences that of the other language.

In order to investigate this, we recruited native Chinese students who were highly proficient in English and spoke Mandarin Chinese (hereafter called Chinese) as their L1. Using the masked priming paradigm, we investigated what the proximate unit was for each of their languages and whether an influence of language regarding the unit-to-frame association process could be found. If the production system mainly uses a Chinese-based (L1) proximate unit irrespective of target languages, no onset priming will be observed when bilinguals are engaged in the task in English (L2). If the production system has discrete proximate units between Chinese and English, then the bilinguals would show syllable but not onset priming in Chinese, while showing onset priming in English. Lastly, if proficiency in English influences Chinese word production, we may expect onset priming even in Chinese.

## Method

### Participants

Twenty Chinese native participants (attending various universities in the Beijing area, China, 12 female, age 24.2±2.9 years) were recruited to take part in this experiment. Participants received 50 RMB (≈8 US $) compensation. The current study was approved by the ethics committee of the Institute of Psychology, Chinese Academy of Sciences (Beijing). Written consent was obtained from participants before the experiment started. Half of the participants passed the Test for English Majors at the highest level (TEM-8). The rest passed either the College English Test (CET-6) or the TOEFL/IELTS exams. Participants reported to have started to come in contact with English around 11.3±2.5 years of age. Active English ability (speaking/writing) self-report was 6.8±1.2 on a 1–10 scale and passive ability (reading/listening) was self-reported to be 7.6±1.0. Average high-school grade was 8.1±1.4 on a 1–10 scale and participants reported that their amount of speaking, reading and listening (in hours) per day on average was: 0.4 h±0.3, 1.9 h±1.4 and 1.0 h±0.8 respectively, also they obtained a very high total (4847±342; score range 0–5000) on the Swansea English vocabulary test we administered after the experiment (for more details see Meara [18]). All participants had normal or corrected-to-normal vision.

### Stimuli

#### English Experiment

Forty-two target words were selected (half mono-, half bi-syllabic). The log KF frequency was 9.6±1.2 per million (taken from the English Lexicon Project [19]), length was 5.1±0.9 letters and syllable length was 1.5±0.5 syllables. The following priming conditions were created: Identity (prime and target are identical), Onset Overlap – primes had a single phoneme overlap with the target – vs. their Onset Control (e.g. target BENCH, primes: bark vs. dark), CV Overlap – primes had onset+vowel overlap with the target – vs. their CV Control (e.g. target BENCH, primes: bell vs. cell). There was no statistical difference between the four prime types, i.e. Onset Overlap, Onset Control, CV Overlap, CV Control in terms of frequency (*F*<1) and length (*F*<1; all groups had one syllable), and when Identity (prime = target) was included in the comparison, there was again no difference in frequency (*F*<1), but there was a difference between identity and the other primes in terms of length, *F*(4) = 48.4, *p*<.001, and syllables, *F*(4) = 45.1, *p*<.001, with the identity being 1.4 phoneme longer and having half a syllable extra compared to the other groups. See Table 1 for stimulus properties and Appendix S1 for an overview.

**Table 1:** Properties of English Prime Words (examples are for the target: BENCH).

Prime Type	Log Frequency (SD)	Length (SD)	Syllables (SD)
Identity (bench)	9.6 (1.2)	5.1 (0.9)	1.5 (0.5)
Onset Overlap (bark)	9.9 (1.3)	3.6 (0.5)	1 (0)
Onset Control (dark)	9.5 (1.3)	3.6 (0.5)	1 (0)
CV Overlap (bell)	9.5 (1.4)	3.8 (0.4)	1 (0)
CV Control (cell)	9.6 (1.2)	3.8 (0.4)	1 (0)

Note: Target properties are identical to Identity Prime.

#### Chinese Experiment

Twenty-four target words were selected; half of them adhered to a CV (e.g. /ba/) and the other half to a CVN (e.g. /ban/) structure, average log frequency was 5.0±1.5, taken from [20] and strokes were on average 8.0±3.1. Primes were selected to match syllabic structure, that is: Same Syllabic Structure (i.e. CV-CV or CVN-CVN) or Different Syllabic Structure (i.e. CVN-CV or CV-CVN) and the amount of overlap (Onset, CV/CVN and Control). Stimuli were controlled for log frequency (Onset Overlap = 4.4±2.1, CV/CVN Overlap = 4.4±2.0, Control = 4.8±1.7, *F*<1), strokes (Onset Overlap = 10.0±3.2, CV/CVN Overlap = 10.4±3.8, Control = 9.6±3.5, *F*<1). We also made sure to avoid characters with multiple pronunciations and/or semantic or radical overlap, see Appendix S2 for an overview.

### Design

#### English Experiment

The design adopted a 5 (Repetition: 1, 2, 3, 4, 5)×5 (Prime Type: Identity, Onset Overlap, Onset Control, CV Overlap, CV Control) within participants design.

#### Chinese Experiment

The experiment adopted a 2 (Syllabic Structure: Same, Different)×3 (Overlap: Onset, CV/CVN, Control) within-participants design. For instance, the target ? /ba1/ ‘eight’ was preceded by 1) a same structure onset overlap prime, e.g. ? /bi1/ ‘to force’, 2) a same structure CV/CVN overlap prime, e.g. ? /ba1/ ‘desire’, 3) a same structure control prime, e.g. ? /pa1/ ‘lean, bend over’, 4) a different structure onset overlap prime, e.g. ? /bin1/ ‘guest’, 5) a different structure CV/CVN overlap prime, e.g. ? /ban1/ ‘class’, and lastly 6) a different structure control prime, e.g. ? /pan1/ ‘climb’.

To avoid excessive repetitions, prime-target combinations were distributed over participants in a Latin-square design such that each participant named each target only three times (instead of six; i.e. 72 trials overall per participant) though still having encountered all prime types/conditions equally often. Each target occurrence was set in one block, resulting in three blocks in total (with short breaks of 30 seconds between each block and warm-up of three items per block). The order of target words within a block and the block order itself were randomized for each participant.

### Procedure

The experiment was performed using E-Prime 2 Professional Software (Psychology Software Tools). Participants were seated in a quiet room approximately 70 cm from a 21 inch CRT computer screen with a refresh rate of 100 Hz. Naming latencies were measured from target onset using a voice-key, connected with the computer via a PST Serial Response Box.

Each trial involved the following sequence: a fixation cross (+) was presented at the center of the screen for 1,000 ms, followed by a forward mask (##) for 500 ms, subsequently the prime was presented for 50 ms, followed by the target word which disappeared after 2 seconds or when participants made a vocal response. Primes, masks and targets were presented in 28-point boldfaced Song or Courier New font. The visual angles of the target words were less than 2 degrees both horizontally and vertically. Participants were asked to name the word aloud as quickly and accurately as possible. Following each response, the experimenter judged whether the response was correct or not (or whether a voice key error had occurred). Both experiments took less than 10 minutes each.

## Results

### English Experiment

In total, ten percent of the data were discarded comprising target words which were incorrectly pronounced (coded by experimenter during the experiment) and/or target words which were unfamiliar to the participant (assessed by a post-test questionnaire) totaling 4.9%. Furthermore, voice-key errors (e.g. disfluencies, accidental triggering) and outliers (set to 2 SD of a participant's mean per condition per repetition) amounted to 5.1% of the data being discarded, see Table 2 for results.

**Table 2:** Results for the English part of the experiment (RT = average reaction time; SD = standard deviation; %E = percentage error).

Prime Type	Example	RT (SD)	%E
Identity	bench - BENCH	618 (86)	4.6
Onset Overlap	bark - BENCH	653 (77)	4.9
Onset Control	dark - BENCH	675 (89)	5.5
CV Overlap	bell - BENCH	646 (83)	4.9
CV Control	cell - BENCH	677 (93)	4.6

There was a main effect of Repetition, indicating that subjects responded faster with increasing repetitions, *F_1_*(4, 76) = 57.1, *MS_e_* = 6198.4, *p*<.001; *F_2_*(4, 156) = 106.7, *MS_e_* = 6999.6, *p*<.001; *Min F′* (4, 157) = 37.2, *p*<.001. There was a main effect of Prime Type, *F_1_*(4, 76) = 38.8, *MS_e_* = 1573.8, *p*<.001; *F_2_*(4, 156) = 31.4, *MSe* = 3714.7, *p*<.001; *Min F′* (4, 218) = 17.4, *p*<.001. Importantly, there was *no* interaction between Repetition and Prime Type, both *F*s<1. Planned comparisons showed that targets were named significantly faster in the Identity condition (bench – BENCH) than any of the other prime type conditions (all *ps*<.001). Furthermore, When the onset overlapped (i.e., bark - BENCH), there was a 22 ms significant facilitation effect, *t_1_*(19) = 3.7, *SD* = 27.0, *p*<.001; *t_2_*(41) = 4.1, *SD* = 36.8, *p*<.001, *Min F′* (1, 48) = 7.5, *p*<.01, compared to its control. In addition, when the CV overlapped (i.e., bell – BENCH), there was a 31 ms facilitation effect, *t_1_*(19) = 5.8, *SD* = 23.8, *p*<.001; *t_2_*(41) = 4.7, *SD* = 40.6, *p*<.001, *Min F′* (1, 58) = 13.3, *p*<.001, compared to its control. The 7 ms difference between Onset- and CV Overlap was not significant, *t_1_*(19) = 1.3, *SD* = 21.9, *p* = .194; *t_2_*(41) = 1.3, *SD* = 31.7, *p* = .186, *Min F′* (1, 52) = .85, *p* = .362, indicating that the facilitation effects are mainly due to the initial phoneme overlap (see also Kinoshita, 2000). The error rates did not reveal a significant effect of Prime Type condition (*Fs*<1) and were therefore not further analyzed.

### Chinese Experiment

In total, 7.2% percent of the data were discarded, 0.6% due to errors (e.g. non-correct target naming) additionally voice-key errors (e.g. disfluencies, accidental triggering) and outliers (set to 2 SD of a participant's mean per condition) amounted to 6.6% of the data being discarded, see Table 3 for results. There was no main effect of Syllabic Structure (same, different), both *F*s<1. There was a main effect of Prime Type (onset, CV/CVN, control), *F_1_*(2, 38) = 14.0, *MS_e_* = 301.7, *p*<.001, *F_2_*(2, 46) = 3.1, *MS_e_* = 1854.2, *p* = .05, *min F′*(2, 65) = 2.54, *p* = .09, and there was an interaction between Syllabic Structure and Prime Type in the subjects, *F_1_*(2, 38) = 5.3, *MS_e_* = 213.5, *p*<.01, but not the items analysis, *F_2_*<1. Planned comparisons showed that there was significant priming for the Onset condition when the syllabic structure overlapped, *t_1_*(19) = 5.2, *SD* = 22.7, *p*<.001; *t_2_*(23) = 2.5, *SD* = 50.0, *p*<.05, *min F′*(1, 33) = 5.1, *p*<.05; and also for the CV/CVN, *t_1_*(19) = 5.3, *SD* = 23.0, *p*<.001; *t_2_*(23) = 2.7, *SD* = 48.1, *p*<.05, *min F′*(1, 34) = 5.8, *p*<.05. However, these effects were not consistent when the syllabic structure did not overlap, onset prime vs. control, *t_1_*(19) = 2.1, *SD* = 19.8, *p*<.05; *t_2_*(23) = 1.2, *SD* = 53.7, *p* = .25, *min F′*(1, 36) = 1.1, *p* = .30 and CV prime vs. control, *t_1_*(19) = 1.7, *SD* = 21.2, *p* = .11, *t_2_*(23) = 1.0, *SD* = 54.7, *p* = .30, *min F′*(1, 36) = .74, *p* = .39. The error rates were low and did not reveal a significant effect of prime type condition (*Fs*<1) and were therefore not further analyzed.

**Table 3:** Results for Chinese Experiment (RT = average reaction time; SD = standard deviation; %E = percentage error).

Prime Type	Examples (prime - target)	RT (SD)	%E
Same Syllabic Structure			
Onset	/bi1/ - /ba1/ or /bin1/ - /ban1/	543 (71)	0.4
CV/CVN	/ba1/ - /ba1/ or /ban1/ - /ban1/	541 (59)	0.0
Control	/pa1/ - /ba1/ or /pan1/ - /ban1/	569 (71)	0.0
Different Syllabic Structure			
Onset	/bin1/ - /ba1/ or /bi1/ - /ban1/	549 (72)	0.8
CV	/ban1/ - /ba1/ or /ba1/ - /ban1/	551 (68)	1.3
Control	/pa1/ - /ban1/ or /pan1/ - /ba1/	559 (70)	0.8

## Discussion

The data clearly show onset, priming for high proficient bilingual Chinese speakers in their L2 (English). Apparently, in these bilingual speakers, the phoneme is a unit which can be primed. This indicates that in their L2 their phonology is assembled similarly to English native speakers. If it were assembled in an L1-Chinese (syllabic) way [10], [11] sub-syllabic priming effects would have been unlikely to emerge. Our bilinguals also showed reliable syllable priming effect in Chinese, replicating previous studies testing monolingual Chinese speakers [21]. In addition, we found sub-syllabic priming effects *even in Mandarin Chinese*, although these effects were observed only when the *syllabic structure of prime-target pairs* overlapped.

In light of these results it is important to mention that empirical observations by Wong and Chen [16], [17] who used the picture naming paradigm as well as Wong et al. [12] who used implicit priming, indicated the existence of sub-syllabic priming in Cantonese Chinese (see introduction). However, as participants in all three studies were living in Hong Kong and were recruited from the Chinese University of Hong Kong and Hong Kong University (all highly bilingual environments) it may have been possible that their L2-English proficiency was considerable (A.W. Wong, personal communication, March 5, 2013). This, according to the findings reported in the current paper, could have had an influence on how phonological information was processed in their L1 (Cantonese). Future work investigating the proximate phonological unit in Cantonese with high- and low-proficient L2-English speakers should be able to assess whether Cantonese shows the same pattern of results as Mandarin does or whether sub-syllabic effects appear in Cantonese regardless of any L2 proficiency. In addition, we suggest that studies exploring the proximate unit should investigate whether proficiency in other languages exists for participants, even when reporting findings for the L1.

Recently, sub-syllabic priming in Chinese has also been investigated by Qu, Damian, and Kazanina [22] who suggest a functional role of *phonemes* at the stage of phonological encoding in Mandarin Chinese. Qu et al. asked participants to name a picture including its color (for example: “yellow box” /huang2he2zi/) while recording participants' event related potentials (ERPs). Yellow (/huang2/) and “box” (/he2zi/) overlapped in their onset, while green box (/lü4he2zi/) did not. Consistent with earlier findings by Chen et al. [10] and O'Seaghdha et al. [11], Qu et al. [22] did not observe a behavioral difference in RTs for onset repetition but they did observe more negative ERP amplitudes. They attributed this to a higher load for internal speech monitoring due to onset repetition; which would cancel out onset facilitation (p. 14268). However, on the basis of our results one may speculate whether priming could be contingent on structure overlap in Chinese. For instance, most of Qu et al.'s stimuli (e.g. /huang2/ and /he2/) do not share structure which might have some influence regarding the absence of a behavioral effect. However, Chen et al. [10] (p. 779) and O'Seaghdha et al. [11] (p. 300) *did* use similarly structured stimuli (e.g. CV starting with “mo, ma, mu, mi”) and did not find onset priming as well. Our findings are therefore most likely the result of high L2-proficiency although more work is necessary to investigate why the L1 sub-syllabic priming reported in this paper was obtained only when the structure overlapped.

Note that our L1 sub-syllabic findings do not have to be incompatible with the role of the syllable as proximate unit proposed by O'Seaghdha et al. [11]. For instance, Qu et al. [22] (p. 14268) also state that syllables are the primary selectable phonological units below the word level and phonemic segments may be retrieved via *syllabic mediation*. In addition, O'Seaghdha, Chen & Chen [23] and O'Seaghdha et al. [11] indicate that their model for Mandarin Chinese assumes that, although syllables are the primary (first selected) unit for language production, importantly, *phonemic specification* does occur for selected syllables.

Before reaching any conclusions, two possible considerations regarding our study should be discussed. The first one is that the Chinese part of the experiment always followed the English experiment. We therefore cannot rule out any spillover as a result of experiment order. However, as this was a masked priming study, participants were not consciously aware of the prime-target relationship and the effect is therefore unlikely to have been strategic. However, even if order had an effect, this does not compromise our conclusion that the assembly of phonology in Chinese can be affected by the use of English in our participants.

The second is that the English uses a script, in which orthographic units are used that roughly correspond to phonemes, whereas Chinese script is syllabic. The same concern was put forward by Verdonschot et al. [14], who conducted two priming experiments in Japanese, in which they changed the script type to romaji (alphabetic Japanese). Importantly, they essentially found the same results (only mora priming in Japanese) regardless of script type. In addition, Chen and Chen [24] in two experiments using auditory and visual implicit priming and picture naming showed that syllabic overlap but never phonemic overlap produced significant priming regardless whether prompts were written, spoken or pictorial. Chen and Chen's study [24] suggest that syllabic priming in Chinese is therefore not due to the properties of Chinese script. Based on the results of Verdonschot et al. [14] and Chen and Chen [24], it is reasonable to assume that the size of the unit to fill the metrical frames is not dependent on the properties of script per se.

In conclusion, our data showed onset priming in English for highly proficient Chinese-English bilinguals. Clearly, their proximate unit is phonemic in nature when speaking in their L2. Our data also indicate that proficiency in English has an effect on the processing of their native language, Chinese, although the significant sub-syllabic priming effects were confined to situations in which the syllabic structure of prime and target overlap (i.e. CV-CV or CVN-CVN). Future work should investigate whether for instance low proficient Chinese – English bilinguals also show onset effects in their L2/L1. Although the syllable still plays the main role in constructing Mandarin Chinese phonology [11], [21], [25] this study does report sub-syllabic priming for Chinese as a consequence of having a good command of English, although this only occurred when the syllabic structure overlaps.

Finally, we would like to advocate that future models of language production should allow for different grain size of Proximate Phonological Units and influences between various languages in their account(s) of how the unit-to-frame association process to construct phonology takes place.

## Supporting Information

Appendix S1 English Stimuli (Experiment 1).
(PDF)

Appendix S2 Chinese Stimuli (Experiment 2).
(PDF)
