# Supplementary material for: The Proximate Phonological Unit of Chinese-English Bilinguals: Proficiency Matters
Source: PLoS One. 2013 Apr 30;8(4):e61454. doi: 10.1371/journal.pone.0061454 (PMC3640013; doi:10.1371/journal.pone.0061454)
Supplement: Appendix S1 — English Stimuli (Experiment 1). (PDF) [file pone.0061454.s001.pdf]

S1 Appendix – A – English Stimuli (Experiment 1)

Target	Prime Type				
	Identity	Onset Overlap	Onset Control	CV Overlap	CV Control
BENCH	bench	bark	dark	bell	cell
BITTER	bitter	bank	tank	bill	hill
BOTTLE	bottle	bat	rat	box	fox
BUDGET	budget	boom	zoom	bump	jump
BUDGE	budge	boat	coat	bust	lust
DESK	desk	dust	rust	dead	head
DEPTH	depth	dock	rock	deck	peck
DANCE	dance	deal	seal	dad	bad
FABRIC	fabric	feed	seed	fan	ban
FANCY	fancy	fold	bold	fat	mat
FERRY	ferry	fire	hire	fence	hence
FISH	fish	fake	lake	fit	hit
FINGER	finger	folk	yolk	fix	mix
GANG	gang	gold	mold	gap	lap
HANDLE	handle	heal	meal	hack	lack
HEAVY	heavy	hide	ride	hell	yell
JACKET	jacket	joy	boy	jam	ham
LEVER	lever	law	saw	leg	beg
LETTER	letter	lint	hint	lend	bend
LIQUID	liquid	loss	moss	lip	hip
LISTEN	listen	lone	tone	link	wink
LOCK	lock	lag	tag	lot	pot
LUNCH	lunch	list	mist	luck	duck
MATCH	match	mop	top	mad	pad
MAGNET	magnet	mean	bean	map	rap
MIDDLE	middle	mark	park	mill	pill
MINT	mint	mall	fall	miss	kiss
MONSTER	monster	math	path	mob	rob
PANTS	pants	pea	tea	pack	tack
PITCH	pitch	pool	cool	pink	sink
PILLOW	pillow	poke	joke	pick	kick
RAPID	rapid	roll	poll	rack	sack
RISK	risk	rain	pain	rip	dip
RICH	rich	rub	pub	ring	sing
SEVEN	seven	sit	kit	set	let
SINGLE	single	save	cave	silk	milk
SICK	sick	sand	band	sip	tip
TEST	test	tall	wall	tell	fell
TEXT	text	toy	soy	tend	send
TAXI	taxi	tool	fool	task	mask
TICK	tick	toss	boss	till	fill
WISH	wish	wax	tax	wit	bit
