# Supplementary material for: The Proximate Phonological Unit of Chinese-English Bilinguals: Proficiency Matters
Source: PLoS One. 2013 Apr 30;8(4):e61454. doi: 10.1371/journal.pone.0061454 (PMC3640013; doi:10.1371/journal.pone.0061454)
Supplement: Appendix S2 — Chinese Stimuli (Experiment 2). (PDF) [file pone.0061454.s002.pdf]

S2 Appendix B – Chinese Stimuli (Experiment 2)

Target	Prime Type					
	Same-Syllable Structure (SS)			Different Syllable Structure (DS)		
	Onset	CV/CVN	Control	Onset	CV	Control
ba1 八	bi1 逼	ba1 巴	pa1 趴	bin1 宾	ban1 班	pan1 攀
ban1 搬	bin1 宾	ban1 班	pan1 攀	bi1 逼	ba1 巴	pa1 趴
hu1 乎	ha1 哈	hu1 忽	tu1 突	han1 憨	hun1 荤	tun1 吞
hun1 昏	han1 憨	hun1 荤	tun1 吞	ha1 哈	hu1 忽	tu1 突
sha1 杀	she1 奢	sha1 沙	cha1 插	shen1 身	shan1 删	chan1 搀
shan1 山	shen1 身	shan1 删	chan1 搀	she1 奢	sha1 沙	cha1 插
xi1 西	xu1 需	Xi1 息	qi1 七	xun1 熏	xin1 新	qin1 亲
xin1 心	xun1 熏	xin1 新	qin1 亲	xu1 需	xi1 息	qi1 七
ya1 鸭	yu1 迂	ya1 压	ta1 他	yun1 晕	yan1 淹	tan1 贪
yan1 烟	yun1 晕	yan1 淹	tan1 贪	yu1 迂	ya1 压	ta1 他
cha2 茶	chu2 除	cha2 察	na2 拿	chun2 纯	chan2 馋	nan2 男
chan2 缠	chun2 纯	chan2 馋	nan2 男	chu2 除	cha2 察	na2 拿
pi2 皮	pa2 爬	pi2 啤	mi2 迷	pan2 盘	pin2 频	min2 民
pin2 贫	pan2 盘	pin2 频	min2 民	pa2 爬	pi2 啤	mi2 迷
qi2 奇	qu2 渠	qi2 其	li2 离	qun2 群	qin2 琴	lin2 林
qin2 秦	qun2 群	qin2 琴	lin2 林	qu2 渠	qi2 其	li2 离
zhe3 者	zhu3 煮	zhe3 褶	re3 惹	zhun3 准	zhen3 诊	ren3 忍
zhen3 枕	zhun3 准	zhen3 诊	ren3 忍	zhu3 煮	zhe3 褶	re3 惹
ba4 罢	bi4 必	ba4 霸	ta4 踏	bin4 殡	ban4 办	tan4 探
ban4 半	bin4 殡	ban4 办	tan4 探	bi4 必	ba4 霸	ta4 踏
du4 肚	da4 大	du4 渡	gu4 顾	dan4 蛋	dun4 盾	gun4 棍
dun4 顿	dan4 蛋	dun4 盾	gun4 棍	da4 大	du4 渡	gu4 顾
la4 辣	lu4 鹿	la4 腊	ma4 骂	lun4 论	lan4 滥	man4 慢
lan4 烂	lun4 论	lan4 滥	man4 慢	lu4 鹿	la4 腊	ma4 骂
